# When Quality Beats Quantity: Decision Theory, Drug Discovery, and the Reproducibility Crisis

**DOI:** 10.1371/journal.pone.0147215

**Published:** 2016-02-10

**Authors:** Jack W. Scannell, Jim Bosley

**Affiliations:** 1 The Centre for the Advancement of Sustainable Medical Innovation, University of Oxford, Oxford, United Kingdom; 2 Innogen Institute, Science, Technology and Innovation Studies, University of Edinburgh, Edinburgh, United Kingdom; 3 J W Scannell Analytics Ltd., 32 Queen’s Crescent, Edinburgh, United Kingdom; 4 Clerbos LLC, Kennett Square, Pennsylvania, United States of America; Politecnico di Torino, ITALY

## Abstract

A striking contrast runs through the last 60 years of biopharmaceutical discovery, research, and development. Huge scientific and technological gains should have increased the quality of academic science and raised industrial R&D efficiency. However, academia faces a "reproducibility crisis"; inflation-adjusted industrial R&D costs per novel drug increased nearly 100 fold between 1950 and 2010; and drugs are more likely to fail in clinical development today than in the 1970s. The contrast is explicable only if powerful headwinds reversed the gains and/or if many "gains" have proved illusory. However, discussions of reproducibility and R&D productivity rarely address this point explicitly. The main objectives of the primary research in this paper are: (a) to provide quantitatively and historically plausible explanations of the contrast; and (b) identify factors to which R&D efficiency is sensitive. We present a quantitative decision-theoretic model of the R&D process. The model represents therapeutic candidates (e.g., putative drug targets, molecules in a screening library, etc.) within a “measurement space", with candidates' positions determined by their performance on a variety of assays (e.g., binding affinity, toxicity, *in vivo* efficacy, etc.) whose results correlate to a greater or lesser degree. We apply decision rules to segment the space, and assess the probability of correct R&D decisions. We find that when searching for rare positives (e.g., candidates that will successfully complete clinical development), changes in the predictive validity of screening and disease models that many people working in drug discovery would regard as small and/or unknowable (i.e., an 0.1 absolute change in correlation coefficient between model output and clinical outcomes in man) can offset large (e.g., 10 fold, even 100 fold) changes in models’ brute-force efficiency. We also show how validity and reproducibility correlate across a population of simulated screening and disease models. We hypothesize that screening and disease models with high predictive validity are more likely to yield good answers and good treatments, so tend to render themselves and their diseases academically and commercially redundant. Perhaps there has also been too much enthusiasm for reductionist molecular models which have insufficient predictive validity. Thus we hypothesize that the average predictive validity of the stock of academically and industrially "interesting" screening and disease models has declined over time, with even small falls able to offset large gains in scientific knowledge and brute-force efficiency. The rate of creation of valid screening and disease models may be the major constraint on R&D productivity.

## Introduction

The scope, quality and cost efficiency of the scientific and technological tools that are widely believed to be important for progress in biopharmaceutical discovery and research have improved spectacularly. To quote a review from 2012 [1]: *“*… *combinatorial chemistry increased the number of drug-like molecules that could be synthesized per chemist per year by perhaps 800 times through the 1980s and 1990s* [2] [3] [4] *and greatly increased the size of chemical libraries* [5]. *DNA sequencing has become over a billion times faster since the first genome sequences were determined in the 1970s* [6] [7] *aiding the identification of new drug targets*. *It now takes at least three orders of magnitude fewer man-hours to calculate three-dimensional protein structure via x-ray crystallography than it did 50 years ago* [8] [9], *and databases of three-dimensional protein structure have 300 times more entries than they did 25 years ago* [10] [9], *facilitating the identification of improved lead compounds through structure-guided strategies*. *High throughput screening (HTS) has resulted in a tenfold reduction in the cost of testing compound libraries against protein targets since the mid-1990s* [11]. *Added to this are new inventions (such as the entire field of biotechnology*, *computational drug design and screening*, *and transgenic mice) and advances in scientific knowledge (such as an understanding of disease mechanisms*, *new drug targets*, *biomarkers*, *and surrogate endpoints)*.*”*

These kinds of improvements should have allowed larger biological and chemical spaces to be searched for therapeutic conjunctions with ever higher reliability and reproducibility, and at lower unit cost. That is, after all, why many of the improvements were funded in the first place. However, in contrast [12], many results derived with today’s powerful tools appear irreproducible[13] [14][15] [16]; today’s drug candidates are more likely to fail in clinical trials than those in the 1970s [17] [18]; R&D costs per drug approved roughly doubled every ~9 years between 1950 and 2010 [19] [20] [1], with costs dominated by the cost of failures [21]; and some now even doubt the economic viability of R&D in much of the drug industry [22] [23].

The contrasts [12] between huge gains in input efficiency and quality, on one hand, and a reproducibility crisis and a trend towards uneconomic industrial R&D on the other, are only explicable if powerful headwinds have outweighed the gains [1], or if many of the “gains” have been illusory [24] [25] [26].

We believe that a variety of standard tools from the fields of decision theory and decision analysis (DT) [27] [28] [29] [30] [31] shed light on the headwinds and may help distinguish the kind of gains that are likely to be real. The Methods and Results section of the paper presents a DT-based model of biopharmaceutical R&D and quantitative analyses that explore the factors to which R&D decisions are sensitive. The model is described in terms of commercial R&D, but we think the framework and the results are generalizable to the academic setting, and to “translation” from academia to industry; in fact to many situations where positives (e.g., good drug targets, good candidate therapeutic mechanisms) are rare and where a large universe of possibilities is filtered via a series of measurements and decisions to a small set of possibilities. In statistical or DT terms, the mechanics of the model are fairly standard. The model is a classifier in the presence of multiple, or multistep, predictors. However, the application is, we think, novel.

Readers who are less familiar with statistics and DT may prefer to read the Discussion section before returning to the Methods and Results. The Discussion is in three parts. Part 1 frames headwinds to R&D productivity in terms of the progressive exploitation, exhaustion, and abandonment of disease models with high predictive validity (PV). Part 2 considers the reproducibility crisis in similar terms. Part 3 sets out some practical suggestion to improve PV evaluation and raise PV.

## Methods and Results

### Terminology and Model Structure

**Fig 1:**
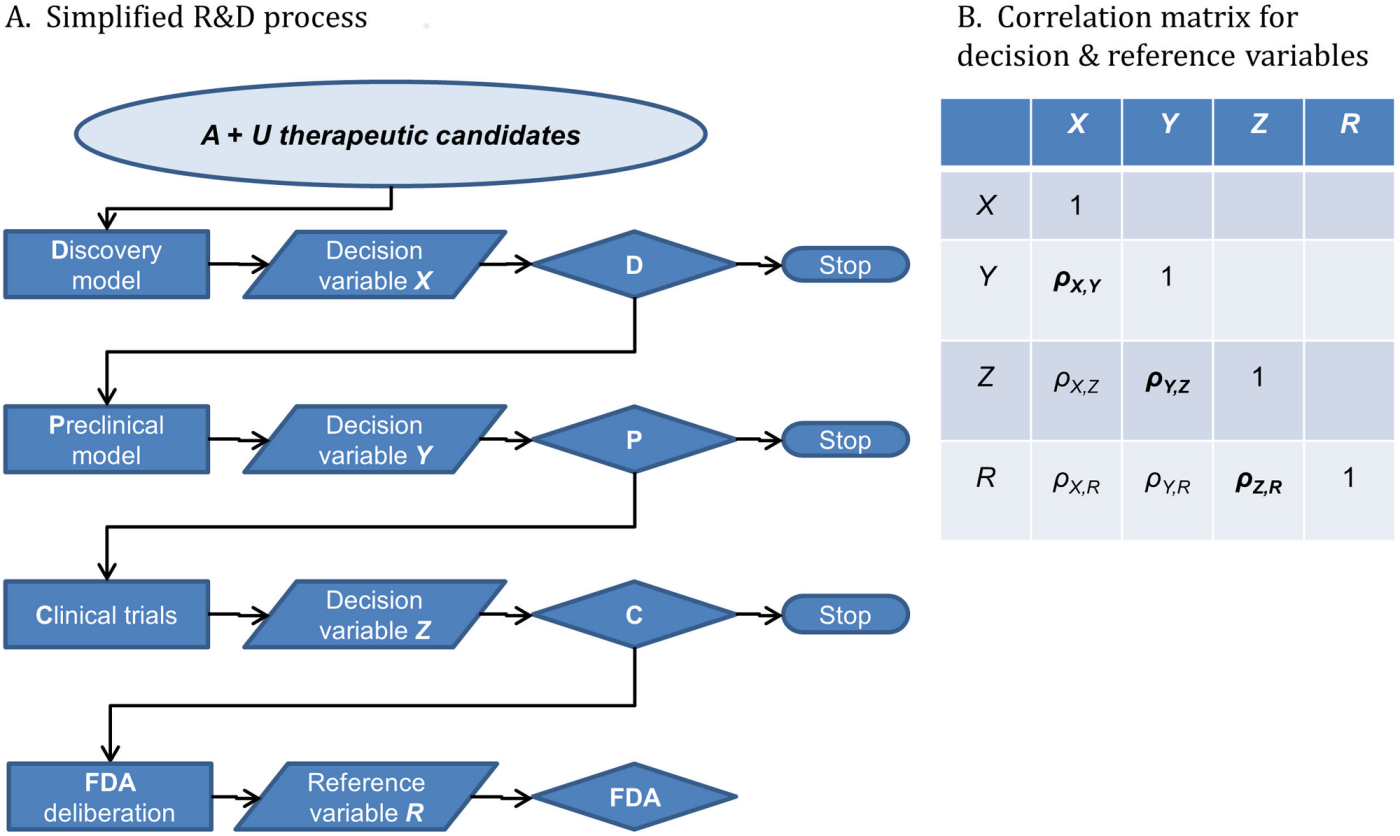
Decision theoretic view of biopharma discovery, research, and development. (A) The process starts with a large set of therapeutic possibilities (light blue oval). These could be putative disease mechanisms or candidate drug targets, in either an academic or commercial setting. However, we discuss them as if they are molecules in a commercial R&D campaign (e.g., compounds in a screening library and the analogues that could be reasonably synthesized to create leads). There are *A* candidates that with perfect R&D decision making and an unlimited R&D budget would eventually be approved by the drug regulator for the indication or indications. There are *U* candidates that would not succeed given similar skill and investment. In general, *U* >> *A*. The Discovery (D), Preclinical (P), and Clinical Trial (C) diamonds are “classifiers” (Table 1). Each takes decision variables (*X*, *Y*, *Z*) from predictive models for some or all of the candidates and tests the variables against a decision threshold, yielding *yeses* which receive further scrutiny or *noes* which are abandoned. The unit cost per surviving candidate increases through the process [21]. Given serial decisions, only *yeses* from C face the gold standard reference test; the drug regulator (e.g., the Food and Drug Administration, or FDA). The other decisions face “imperfect” reference tests [33] [34] [27], the next steps in the process, which are mere proxies for the gold standard. The imperfect reference test for *yeses* from D is provided by P. The imperfect reference test for *yeses* from P is provided by C. (B) Decision variables *X*, *Y*, and *Z*, will correlate to a greater or lesser extent with each other and with the gold standard reference variable *R*. The correlation coefficient between *X* and *Y* is *ρ*_*X*,*Y*_, the correlation coefficient between *Y* and *Z* is *ρ*_*Y*,*Z*_, etc. Most of these correlations will never be measured directly during the R&D process. If *ρ*_*X*,*R*_ is very low, the Discovery stage will not enrich the Preclinical stage for approvable candidates, even if *ρ*_*X*,*Y*_ is high and decisions from D initially appear to have been successful.

We begin by introducing and defining our terms and the basic structure of the model we use to represent the process of discovery, research, and development (Table 1, Fig 1). The code of the programmes that we used to implement our model is in S1 File.

**Table 1:** Decision Theoretic Concepts and Terms [27] [28] [29] [30] [31].

Terms and symbols	Comments
**Decision variable, generally *Y* or *y***	Decision variables are the measures (e.g., binding affinity, IC_50_, C_max_, etc.) on which classification decisions (e.g., go / no-go decisions in R&D) are based. Lower case “*y*” corresponds to specific instances of the decision variable (e.g., *y*_*a*_, *y*_*b*_, *y*_*c*_, etc. as measures for molecules in a sample of drug candidates). Upper case “*Y*” represents the random variable from which specific instances are drawn.
**Reference variable, *R* or *r***	Reference variables provide the test of the performance of the decision process. So, for example, *r*_*a*_ could be efficacy of a specific drug candidate *a* in a Phase III trial that was initiated on the basis of *y*_*a*_ efficacy in a Phase II trial. Upper case “*R*” represents the random variable from which specific instances of are drawn.
**Decision threshold, generally *y***_***t***_	We assume that the decision is “*yes*” when *y* ≥ *y*_*t*_ and “*no*” when *y* < *y*_*t*_ In reality, thresholds may be quantitative or qualitative, implicit or explicit.
**Reference threshold, *r***_***t***_	An item is a positive when *r* ≥ *r*_*t*_ and a negative when *r* < *r*_*t*_.
**Predictive model, PM**	Something that generates decision variables for therapeutic candidates. E.g., Lipinski’s “rule of 5” [32] is a PM of oral bioavailability.
**Predictive validity, PV**	The degree to which the ordering of the population of candidates on the decision variable would match the ordering of the candidates on a corresponding reference variable, in the limit case when sample sizes are large. Here we operationalise PV as the Pearson correlation coefficient between the decision and reference variable. However, it would be reasonable to operationalize PV in other ways (e.g., Spearman’s rank correlation, or area under the ROC curve [27] [28]).
**Reliability**	A variable is reliable if repeat measurements are consistent.
**Classifier**	A process that tests decision variables (e.g., *y*_*a*_, *y*_*b*_,… *y*_*z*_) against a decision threshold, *y*_*t*_, and which returns a “*yes*” when *y* ≥ *y*_*t*_ and “*no*” when *y* < *y*_*t*_. In drug R&D, items that are deemed to be “*yeses*” receive further investment and scrutiny.
**True positives, *TP***	Items classified as *“yes”* which are positive on the basis of the reference variable and reference threshold; when for item *i*, (*y*_*i*_ ≥ *y*_*t*_ and *r*_*i*_ ≥ *r*_*t*_)
**True negatives, *TN***	Items classified as “*no*” which are negative on the basis of the reference variable and reference threshold; when for item *i*, (*y*_*i*_ *< y*_*t*_ and *r*_*i*_ *< r*_*t*_)
**False positives, *FP***	Items classified as “*yes*” but which are negative on the basis of the reference variable and the reference threshold; when (*y*_*i*_ ≥ *y*_*t*_ and *r*_*i*_ *< r*_*t*_)
**False negatives, *FN***	Items classified as “*no*” but which are positive on the basis of the reference variable and the reference threshold; when (*y*_*i*_ *< y*_*t*_ and *r*_*i*_ ≥ *r*_*t*_)
**True positive rate, *TPR***	*TPR = #TP*/(*#TP + #FN*) where *#TP* is the number of true positives, and *#FN* is the number of false negatives
**False positive rate, *FPR***	*FPR = #FP*/(#*FP + #TN*)
**Positive predictive value, *PPV***	*PPV = #TP*/(#*TP +# FP*) = *1 –FDR*
**False discovery rate, *FDR***	*FDR = #FP*/(#*TP + #FP*) = *1 –PPV*
**Number of candidates screened per *TP***	(1 / *TPR*) x [#positives / (#positives + #negatives)]

We note that DT-related ideas, sometimes with a different intellectual heritage, are already applied in many technical activities in drug R&D. For example, they are used extensively with respect to clinical trial design towards the end of the process (e.g., references: [35] [36] [37] [38] [39] [40] [41] [42] [43] [44] [45] [46] [47] [48] [49]), to chemistry and screening near the start of the process (e.g., references: [50] [51] [52] [53] [54] [32] [55] [56] [57] [58] [59] [60]), but more rarely elsewhere (but see, e.g., references: [61] [62] [63]).

A prerequisite for the effective application of DT is the correct representation of the system in which decisions are made [64]. Thus Fig 1B emphasizes the fact that “translational medicine” in general and commercial drug R&D in particular both involve a set of measurements that are often ***intended*** to co-vary or correlate with one another to a greater or lesser degree. The purpose of molecular assays is often to predict *in vivo* potency or toxicity. The purpose of animal efficacy studies is to predict clinical activity in man. This important feature of the process is not captured by some R&D productivity frameworks [21] [61] [65], although it is often reflected in the qualitative discussions that accompany them [21] [65], and is clearly apparent in parts of the clinical trial literature [37] [35] [36]. The frameworks that ignore the correlation between different measures miss, for example, the fact that changing the decision threshold (i.e., stringency), throughput, or model quality at one step in the process implies changes elsewhere in the process (see later).

Also central to our analysis is the concept of “predictive validity” (PV). We define the PV of a decision variable (e.g., a binding affinity measures in a high-throughput drug screen, the “gut feelings” of an expert medicinal chemist, the rank-ordering of drug candidates in an R&D portfolio management meeting, Phase II results, etc.) as the degree to which the ordering of the population of candidates on the decision variable would match the ordering of the candidates on a corresponding reference variable, in the limit case when sample sizes are large. PV is high when there is a high probability that the ordering of drug candidate *a* and drug candidate *b* on the reference variable is the same as the ordering of *a* and *b* on the decision variable (Table 1). The reference variable is the more definitive–and generally more expensive [21]–measure that is made later in the R&D process, with the ultimate “gold standard” reference often being regulatory approval. Note that nearly all decisions in R&D are tested against an “imperfect” reference [33] [34] [27], the next step of the R&D process, and not against “gold standards” such as regulatory approval or therapeutic and commercial success (Fig 1).

We use the term PV because general terms such as “validity” and “validation” have a range of different meanings in the biomedical literature (see, for example: [66] [53] [67] [68] [69] [70] [71]). Our definition of PV also distinguishes PV from reliability (Table 1). Reliability is something that is, in principle at least, amenable to conventional statistical management and can be increased by increasing sample size [29] [13] [72]. While we frame our analyses in terms of PV, one could conduct similar analyses of decision variables’ reliability. We will also use the term predictive model, or PM (Table 1), to refer to a screening or disease model when it is used to generate a decision variable for one or more therapeutic candidates. Again, this is because the term “model” has various different meanings [73] [69].

### The Compounding Effects of True and False Positive Rates

Fig 1A shows a series of decisions acting on an initial sample of therapeutic candidates of which *A* would be approved if fully developed and then scrutinized by the regulator, and of which *U* would not. The objective of the subsequent R&D process is to increase the ratio of approvable to unapprovable candidates.

The ratios of approvable to unapprovable candidates through the process are given by Eqs 1–4. The equations show the importance of the ***spread*** between the *TPR* and *FPR* of each decision, and the ***compounding*** effect of sequential *TPRs* and *FPRs*, in achieving the objective.

$$Q_{\text{start}}=\frac{A}{U}$$ (1)

$$Q_{\text{D}\to \text{P}}=\frac{A}{U}\times \frac{TPR_{\text{D}}}{FPR_{\text{D}}}$$ (2)

$$Q_{\text{P}\to \text{C}}=\frac{A}{U}\times \frac{TPR_{\text{D}}}{FPR_{\text{D}}}\times \frac{TPR_{\text{P}}}{FPR_{\text{P}}}$$ (3)

$$Q_{\text{C}\to \text{FDA}}=\frac{A}{U}\times \frac{TPR_{\text{D}}}{FPR_{\text{D}}}\times \frac{TPR_{\text{P}}}{FPR_{\text{P}}}\times \frac{TPR_{\text{C}}}{FPR_{\text{C}}}$$ (4)

Here, *Q*_start_ is the ratio of approvable to unapprovable candidates in the initial starting set; *Q*_D→P_ is the ratio among candidates leaving Discovery and entering Preclinical; and *Q*_P→C_ is the ratio leaving Preclinical and entering Clinical Trials; etc. *TPR*_D_ and *FPR*_D_ are true and false positive rates for classifier D using the gold standard of regulatory approval (the FDA) as the reference (Fig 1A); *TPR*_P_ and *FPR*_P_ are stepwise true and false positive rates for classifier P using the FDA as the reference; etc.

With a series of high *TPRs* and low *FPRs*, *Q* will tend to be high. With a series of low *TPRs* and high *FPRs*, *Q* will tend to be low. While this is clearly apparent in some R&D productivity analyses [61] [49], the importance of the *TPR* versus *FPR* spread is not captured by other sets of metrics that have been influential in the drug industry[21] [65]. As Cook et al. [65] point out, management metrics that focus on the quantity of R&D activity, not on decision quality, have sometimes proven counterproductive.

Eqs 1–4 also show the importance of starting with the right set of therapeutic candidates (i.e., a sufficiently high *A* to *U* ratio). This topic is already the focus of a large body of literature in, for example, the fields of chemoinformatics, screening library design, and structure-based design, and we do not consider it further in this paper.

### Presentation of the Quantitative Decision Model

We have produced a quantitative decision model that can be applied to the process shown in Fig 1. Each decision or reference variable (the random variables *X*, *Y*, *Z*, …, *R*, Table 1) corresponds to one axis of a multidimensional measurement space. The individual scores of the therapeutic candidates, molecules *a*, *b*, *c*, *d*, etc., on each variable are coordinates in the space. Thus candidate molecule *a* occupies position (*x*_*a*_, *y*_*a*_, *z*_*a*_…), molecule *b* occupies position (*x*_*b*_, *y*_*b*_, *z*_*b*_…), etc. One can apply one or more decision thresholds (thresholds *x*_*t*_, *y*_*t*_, *z*_*t*_, etc.)–or other decision rules–to divide the space and to assess the quantitative relationships between decision performance (e.g., *PPV*, *FDR*, or *TPR*), and a variety of factors such as the proportion of positives at the start of the process (i.e., *A*/(*A* + *U*) in Fig 1), the throughput or brute-force power of each PM, and the degree to which each PM yields decision variables that are correlated with other decision variables and with *R*, the gold standard reference variable (Fig 1B).

For the analyses shown in the body of this paper, the probability density of molecules within the measurement space is a multivariate normal distribution. More formally, we use a random vector of *standardized* covariates **x** = [*X*, *Y*, *Z*, …, *R*] distributed as a multivariate normal distribution, N where *μ* = [0, 0, 0,…, 0] and the covariance matrix, ∑, is equal to the correlation matrix, corr[*X*, *Y*, *Z*, …, *R*]:

x ~N(μ,∑)=N(μ, corr[X,Y,…,R]) (5)

We have repeated the analysis for other probability density functions, with sometimes identical, often similar, but sometimes predictably different results (S2 File).

**Fig 2:**
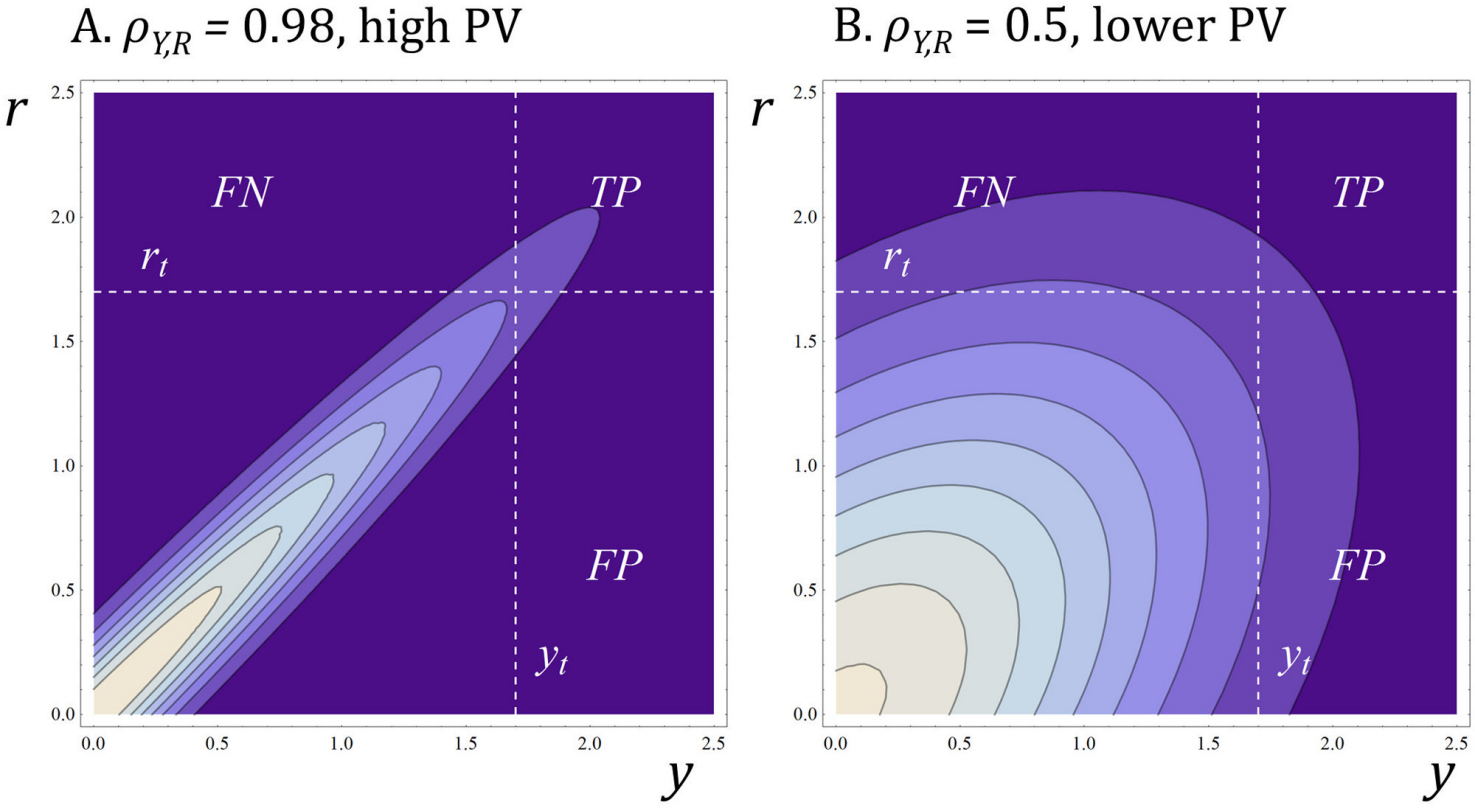
Quantitative classifier model. Bivariate normal probability density function determined by the correlation, *ρ*_*Y*,*R*_, between decision variable, *Y*, and reference variable, *R*. Lighter colours indicate high probability density (candidate molecules more likely to lie here), and darker colours indicate a low probability density (molecules less likely to lie here). The units on the horizontal and vertical axes are one standard deviation. We apply a decision threshold, *y*_*t*_ (vertical dotted line) to the decision variable and then apply a reference test and a reference threshold, *r*_*t*_,(horizontal dotted line) to molecules that exceed the decision threshold *y*_*t*_. In the sensitivity analyses (see later) decision and reference thresholds are varied as is *ρ*_*Y*,*R*_. True positives (*TP*) and false positives (*FP*) correspond to the probability mass in the upper right and lower right quadrants, respectively. (A) When *ρ*_*Y*,*R*_ is high, *PPV* is high. (B) When *ρ*_*Y*,*R*_ is low, *PPV* tends to be low.

The model can be applied to multiple decision variables and classification steps (see later), but we start with a single decision step (Fig 2). Here, the random vector **x** = [*Y*, *R*] is distributed as a bivariate normal distribution, and the correlation coefficient between decision variable *Y* and reference variable *R* is *ρ*_*Y*,*R*_. The correlation parameter, *ρ*_*Y*,*R*_, (Fig 2, Eq 8) operationalises the concept of the predictive validity (PV) of the reference variable. When the correlation between the reference variable and decision variable is high, the ordering of candidates on the decision variable will tend to match the ordering of candidates on the reference variable. It would, of course, be possible to operationalize the concept of PV in other ways (Table 1).

A molecule will be classified as a *yes*, and receive further scrutiny, if its score on the decision variable meets or exceeds a threshold *y*_*t*_ (Fig 2). The decision threshold *y*_*t*_ can be regarded both as a measure of the rate of attrition or stringency of the decision **and also** as a measure of throughput.

This point may not be obvious, but it is important. As *y*_*t*_ rises, fewer candidate molecules are deemed to be *yeses*, so one has to screen more therapeutic candidates for each *yes*. When *y*_*t*_ = 2.32 standard deviation units (horizontal axis, Fig 2), only the top hundredth of molecules will be *yeses*. One would expect to screen one hundred candidates per *yes*. When *y*_*t*_ = 3.09 standard deviation units (Fig 2), only the top thousandth of molecules will be *yeses*. One would expect to screen one thousand molecules per *yes*. Thus, higher decision thresholds depend on higher throughput, and it is higher throughput that makes higher decision thresholds possible.

In some parts of the paper we express stringency or throughput in terms of the probability that a randomly selected candidate lies at or above the decision threshold, *y*_*t*_. This is shown in Eq 6, where Φ is the cumulative distribution function of the standard normal distribution:

$$\text{P}\left(Y\geq y_{t}\right)=1-\Phi \left(y_{t}\right)$$ (6)

To be deemed to be a true positive, a candidate that is a *yes* on the basis of its score on the decision variable must then meet or exceed a threshold *r*_*t*_ on the gold standard reference variable *R*. When *r*_*t*_ is high, fewer candidate molecules within the set that is being searched by the R&D process have the potential to succeed (i.e., *A*/(*A* + *U*) declines as *r*_*t*_ increases). Our definition of *r*_*t*_ is statistical and is not discussed in terms of a specific trial endpoint or experimental outcome. However *r*_*t*_ is realistic in the sense that it will tend to move up and down with common-sense measures of regulatory stringency, or with a common-sense view of the competitive intensity within a therapy area. In some parts of the paper we express the difficulty of the search process in terms of the probability that a randomly selected candidate lies at or above the reference threshold, *r*_*t*_:

$$\text{P}\left(R\geq r_{t}\right)=1-\Phi \left(r_{t}\right)$$ (7)

### Measures of Decision Quality

The proportion of molecules which meets or crosses the decision threshold, *y*_*t*_, and which receives further scrutiny, corresponding to the probability mass to the right of the vertical dotted line in Fig 2, is:

$$\text{P}\left(Y\geq y_{t}\right)=\int_{r=-\infty }^{r=\infty }\int_{y=y_{t}}^{y=\infty }N\left(\mu ,\text{corr}\left[Y,R\right]\right)\;dy\;dr$$ (8)

The proportion of true positives, corresponding to the probability mass in the upper right quadrant of Fig 2, is given by:

$$\text{P}\left(Y\geq y_{t}\text{ and R}\geq r_{t}\right)=\int_{r=r_{t}}^{r=\infty }\int_{y=y_{t}}^{y=\infty }N\left(\mu ,\text{corr}\left[Y,R\right]\right)\;dy\;dr$$ (9)

The proportion of progression decisions which yield true positives is the positive predictive value, or *PPV*. The *PPV* of the classifier is:

$$PPV=\frac{\text{P}\left(Y\geq y_{t}\;and\;R\geq r_{t}\right)}{P\left(Y\geq y_{t}\right)}$$ (10)

*PPV* is an important measure of decision quality in drug R&D because the unit costs per surviving therapeutic candidate tend to rise through the R&D process [21]. Thus, real-world portfolio management processes often seek to maximize *PPV*. Furthermore, *PPV* is equal to (1-*FDR*) where *FDR* is the false discovery rate. Health authorities such as the FDA and the European Medicines Agency (EMA) are often concerned to minimise the *FDR*, which is equivalent to maximising *PPV*.

### A Single Decision Step

**Fig 3:**
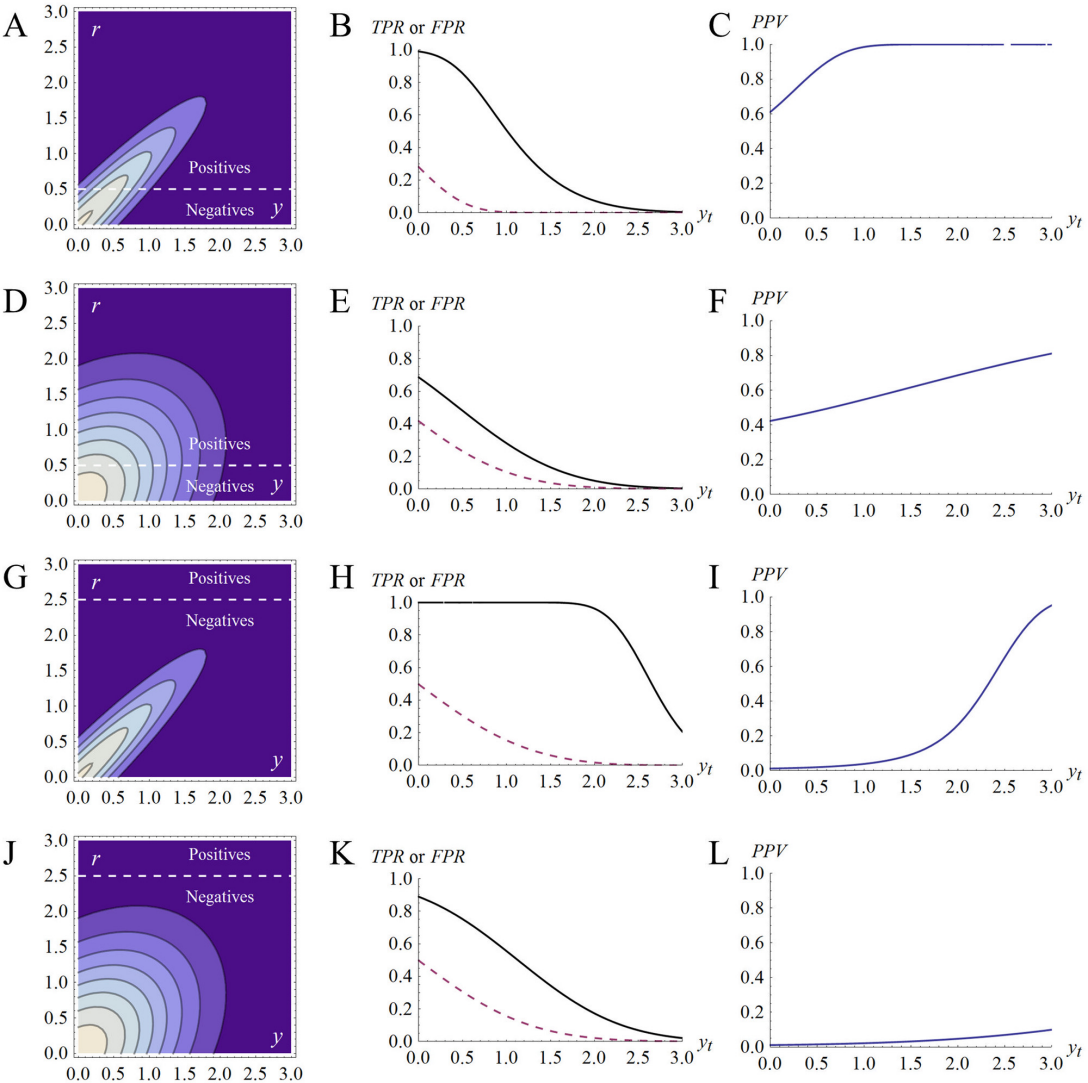
Predictive validity and classifier performance. (A) The bivariate normal probability density function for decision variable *Y* (horizontal axis) and reference variable *R* (vertical axis). The correlation between *Y* and *R* is high (*ρ*_*Y*,*R*_ = 0.95) so the decision variable has high PV. The graph shows only the positive quadrant of the distribution. The reference threshold, expressed here in units of standard deviation, is *r*_*t*_ = 0.5 (dotted line) so positives are common, accounting for P(*R* ≥ *r*_*t*_) ≈ 30% of the probability mass. (B) shows *TPR* (solid line) and *FPR* (dotted line) as the decision threshold, *y*_*t*_, varies. At some thresholds, the spread between the *TPR* and *FPR* is wide. (C) shows *PPV* vs. decision threshold, *y*_*t*_. (D) to (F) repeat the analyses with a decision variable with lower PV (*ρ*_*Y*,*R*_ = 0.4). *PPV* declines vs. panel (C) but *PPV* remains high because positives are common. (G) to (I) repeat that analysis at *ρ*_*Y*,*R*_ = 0.95 but with a high reference threshold (2.5 standard deviation units) and rare positives (P(*R* ≥ *r*_*t*_) ≈ 0.6% of the probability mass). It is possible to achieve a high *PPV*, but only at a high decision threshold when the *TPR* is low, which would require screening a large number of items per positive detected. (J) to (L) show the situation with the same high reference threshold (i.e., rare positives) but with a decision variable with low PV. In this case, *PPV* is low, even with a very high decision threshold and a very low *TPR*.

Fig 3 illustrates of the performance of single decision step. When PV is high, the classifier can effectively distinguish between positives and negatives. When PV is low, it cannot. Fig 3 also illustrates some other typical classifier properties. There is usually a trade-off between *TPR* and *FPR*. When the classifier is stringent (i.e., applies a high decision threshold, which in turn requires a high throughput), the *FPR* tends to be low, but the *TPR* tends to be low too

Fig 3 shows that stringency tends to raise *PPV* (and lower *FDR*), but setting a high decision threshold may not, in practical terms at least, rescue the performance of a classifier if the decision variable has low PV (Fig 3L). A more effective way to tune the decision process to raise parameter *Q*, the ratio of approvable to non approvable candidates at each step (Eqs 1–4), may be to improve the predictive validity of PMs (or to choose therapeutic problems where PV is likely to be high).

Fig 3 also shows that decision performance is sensitive to the reference threshold. When *r*_*t*_ increases and positives become rarer, decision performance tends to becomes worse. Thus, as therapeutic standards within a therapy area rise, a constant set of PMs may appear to perform less well.

### Sensitivity Analysis of a Single Decision Step

**Fig 4:**
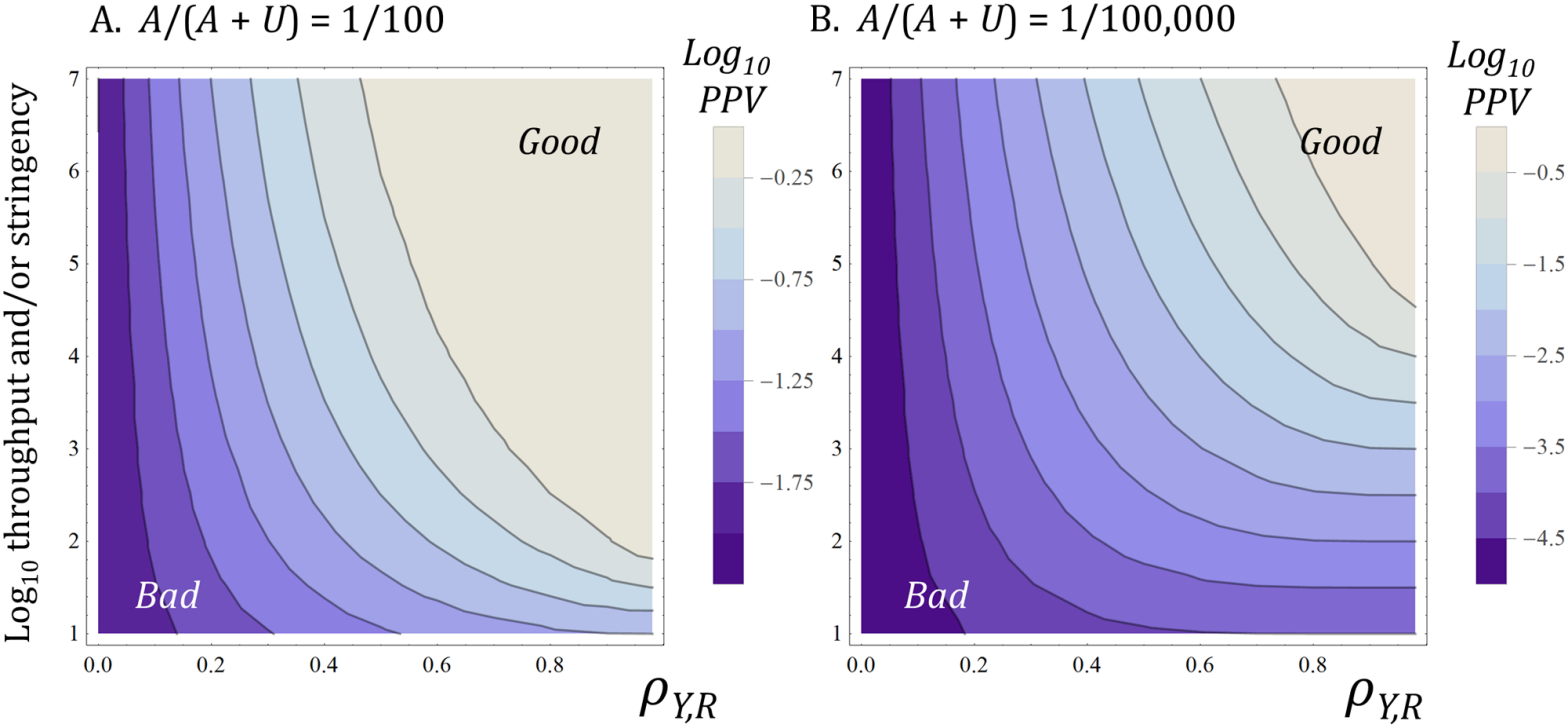
Decision performance as *y*_*t*_ (throughput) and *ρ*_*Y*,*R*_ (predictive validity) vary. Shading shows the *PPV* of the classifier (log_10_ units, with lighter shades showing better performance). The vertical axis represents both decision threshold and screening throughput. The scale is in log_10_ units. 7 represents a throughput of 10^7^ and a decision threshold that accepts only the top 10^7th^ of candidates (P(*Y* ≥ *y*_*t*_) = 10^−7^, Eq 6); 6 represents a throughput of 10^6^ and a decision threshold that accepts only the top 10^6th^ of candidates (P(*Y* ≥ *y*_*t*_) = 10^−6^, Eq 6); etc. The horizontal axis represents PV as the correlation coefficient, *ρ*_*Y*,*R*_, between *Y* and *R*, with the right hand end of each axis representing high PV (*ρ*_*Y*,*R*_ = 0.98), and the left hand end of each axis representing low PV (*ρ*_*Y*,*R*_ = 0). Our choice of scale for each axis is discussed in the main text. In (A), positives are relatively common. Here, P(*R* ≥ *r*_*t*_) = 0.01, or one percent of the candidates entering the classifier. In (B), positives are relatively rare. Here, P(*R* ≥ *r*_*t*_) = 10^−5^, or one hundred thousandth of the candidates entering the classifier. The spacing and orientation of the contours show the degree to which *PPV* changes with throughput and with *ρ*_*Y*,*R*_. *PPV* is relatively sensitive to throughput when *ρ*_*Y*,*R*_ is high and when positives are very rare (lower right hand side of panel B.). However, *PPV* is relatively insensitive to throughput when *ρ*_*Y*,*R*_ is low (left hand side of both panels). For much of the parameter space illustrated, an absolute 0.1 change in *ρ*_*Y*,*R*_ (e.g., from 0.4 to 0.5, or 0.5 to 0.6 on the horizontal axis) has a larger effect on *PPV* than a 10x change in throughput (e.g., from 4 log_10_ units to 5 log_10_ units on the vertical axis).

Fig 4 shows the *PPV* of the classifier as *y*_*t*_ (stringency or throughput) and as *ρ*_*Y*,*R*_ (predictive validity of the decision variable) vary. It shows two conditions, one where the positives are relatively common (P(*R* ≥ *r*_*t*_) = 0.01, or one percent of the candidates entering the classifier) and one where positives are rare (P(*R* ≥ *r*_*t*_) = 10^−5^, or one hundred thousandth of the candidates entering the classifier).

For the single decision step, one can imagine the decision variable, *Y*, as representing an aggregate measure derived from the progressive screening, optimisation, and preclinical assessment of a large number of potential drug candidates. We think such aggregation is reasonable for the purposes of illustration. This is for two reasons. First, the *FPR* and *TPR* of a chain of classifiers are the products of the individual stepwise *FPR*s and *TPR*s (Eqs 1–4). Second, we find similar results for combinations of decision variables across multiple classification steps (see later). Note also that the results we show use parameters that are relevant for discovery and preclinical phases of commercial drug R&D, from which few candidates are selected for clinical trials and from which few randomly selected candidates would succeed in trials (i.e., P(*R* ≥ *r*_*t*_) ≤ 0.1 and P(*Y* ≥ *y*_*t*_) ≤ 0.1). The general model would be applicable to situations where many or even most molecules are positives, in late stage clinical development, for example. However, the quantitative results and conclusions would be different. Furthermore, there is already a mature literature that applies DT-related ideas to clinical development (see, for example: [35] [37] [36] [49])

The scale and range of the vertical axis in Fig 4 can be regarded as representing the range in brute force power or efficiency of PMs in drug R&D. One can conceptualize this in several ways, such as the growth over time in size of compound libraries that can be used in a screening campaign (e.g., from *in vivo* screening in the 1930s to high throughput screening circa 2015), or as the range in the cost efficiency (1/unit cost per therapeutic candidate tested) of PMs today (e.g., from human trials, via *in vivo* primate disease models, via *in vitro* cellular models to *in silico* protein structure based screening) [1] [74].

Several of the results in Fig 4 are unsurprising. First, *PPV* increases as *ρ*_*Y*,*R*_, the correlation between *Y* and *R*, increases. Second, *PPV* increases if one applies very high *y*_*t*_ thresholds (very high throughputs). Third, *PPV* is higher when the reference threshold for positives, *r*_*t*_, is lower. In other words, and rather obviously, there will be a lot of correct decisions to initiate clinical trials when we have PMs with very high PV, which can be reasonably be applied to a very large number of therapeutic candidates, a high proportion of which would have been good enough in the first place to yield successful clinical outcomes.

However, there are results which are less obvious but which appear important for the conduct of decision processes such as drug R&D. The first is the **strength** of the effect of *ρ*_*Y*,*R*_ on *PPV* (see orientation of the *PPV* contours in Fig 4, and note both the logarithmic vertical axis and the logarithmic colour scale). For much of the parameter space illustrated, an absolute 0.1 change in *ρ*_*Y*,*R*_, the correlation coefficient, has a larger effect on *PPV* than a ten-fold or 1 log_10_ unit change in throughput (vertical axis).

We suggest that for many, perhaps most, people working with PMs in drug discovery, an 0.1 absolute change in the correlation between the output of two PMs, or between the decision variable from a PM and the reference variable, would often–even if it were known or knowable–be viewed as small; a difference that would be lost in the general experimental noise. On the other hand, most people would regard a 10 fold increase in throughput or a 10 fold decrease in the unit cost of a PM as a large change.

The second important result is the **interaction** between *y*_*t*_ and *ρ*_*Y*,*R*_ on *PPV* (see how the orientation of the contours changes in Fig 4). Increasing throughput by several orders of magnitude has a **minimal** positive effect on *PPV* when *ρ*_*Y*,*R*_ is very low. Increasing throughput has a large positive effect on *PPV* only when *ρ*_*Y*,*R*_ is high. Modest gains in *ρ*_*Y*,*R*_ can have very large positive effect on *PPV* when throughput is high.

In practical terms, there is little point in investing to increase the throughput of a poor PM or the stringency of the classifier based on that PM. It makes more sense to invest to achieve high PV first. Furthermore, increasing the throughput of a good PM or the speed or stringency of R&D decisions only makes sense if such changes do not cause a meaningful reduction in PV.

### Multiple Decision Steps

With more decision steps, the probability density of candidate molecules within measurement space is determined by the correlation matrix between multiple decision variables, *W*, *X*, *Y*, etc., and the reference variable *R* (Eq 8). Now, the probability that a molecule meets or exceeds a series of decision thresholds on a series of decision variables is given by integrating the probability density function across each variable from the appropriate threshold to infinity (it would, of course, be possible to apply other methods for combining the decision variables, but we do not consider them here). The proportion of true positives when applying 2 decision thresholds, *x*_*t*_ and *y*_*t*_, to two decision variables, *X* and *Y*, corresponds to:

$$\text{P}\left(R\geq r_{t}\text{and}\;X\geq x_{t}\;\text{and}\;Y\geq y_{t}\right)=\int_{r=r_{t}}^{r=\infty }\int_{y=y_{t}}^{y=\infty }\int_{x=x_{t}}^{x=\infty }Ndx\;d\text{y }dr$$ (11)

Note that the single classifier’s quantitative performance depended on only 3 parameters; *y*_*t*_, *r*_*t*_, and *ρ*_*Y*,*R*_. Now with two classifiers and a reference step, there are six parameters. These are the decision and reference thresholds (*x*_*t*_, *y*_*t*_, *r*_*t*_) and three correlation coefficients, one for each unique pairwise correlation; *ρ*_*X*,*Y*_, *ρ*_*X*,*R*_ and *ρ*_*Y*,*R*_. If there are *n* classification steps including the reference test, the number of parameters is given by:

$$\text{number of parameters}=n+\frac{1}{2}(n^{2}-n)$$ (12)

**Fig 5:**
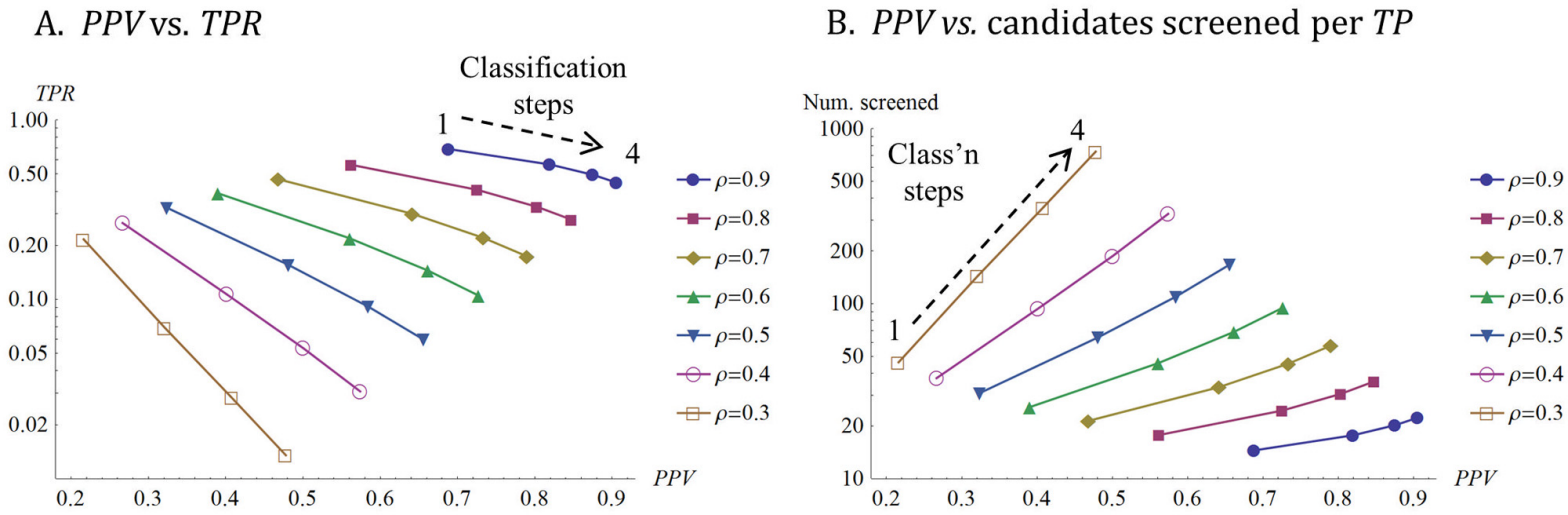
Effect of multiple classification steps. (A) Points represents decision performance with one, two, three, or four, similar classifiers applied in series. Each line represents the same value of correlation coefficient, *ρ*, applied to all pairwise relationships between decision variables and between decision variables and *R*. Thus in each line, all decision variables are equally correlated with each other and with *R*. The correlation coefficient between decision variables (*X*, Y, *W*, *Z*) and *R* vary from 0.9 (high PV, top right line) to 0.3 (low PV, bottom left line). The top left point on each line shows a single classifier applied to *X*, with each additional point towards the bottom and right of each line showing the effects of adding an additional classifier, up to a maximum of 4 classifiers. The top decile of candidates in the starting set exceed each decision threshold and the reference threshold (i.e., P(*X* ≥ *x*_*t*_) = P(*Y* ≥ *y*_*t*_) = P(*W* ≥ *w*_*t*_) = P(*Z* ≥ *z*_*t*_) = P(*R* ≥ *r*_*t*_) = 0.1). In general, adding more steps increases *PPV* but at the cost of a lower *TPR*. There are diminishing returns from each additional classifier, particularly when the decision variables are highly correlated with one another. Furthermore, a single classifier that is highly correlated with *R* (e.g., the uppermost points on the lines with high correlation coefficients) often outperforms a combination of several classifiers with lower correlations with *R* in terms of both *PPV* and *TPR*. Note the logarithmic vertical axis. (B) is exactly as (A) but shows on the vertical axis the number of candidates screened per *TP* (Table 1). The number of candidates that must be screened per true positive identified increases as *ρ* (PV) declines because positives are wrongly rejected. Increasing *ρ* (PV) increases search efficiency. Note the logarithmic vertical axis.

Given the fact that the number of model parameters increases rapidly as the number of decision variables or decision steps increases, we touch on only two relatively simple examples of multiple decision steps here. The first is illustrated in Fig 5, which shows the consequence of stacking a series of similar classifiers. It is possible to increase *PPV* with several similar steps, but at the cost of reducing *TPR*, which means screening more candidates for each positive that the search ultimately yields. As with the single classification step (Figs 2–4) performance can be very sensitive to PV. So, for example, for the parameters shown, one classification step when the correlation between *Y* and *R* is *ρ* = 0.9 outperforms 2 classification steps when the correlations coefficients between *X*, *Y* and *R* are all 0.7 or 3 classification steps when the correlation coefficients are all 0.6. The single step at *ρ* = 0.9 yields both a higher *TPR* and a higher *PPV* having tested far fewer candidates. In this case, the number candidates screened per *TP* (Table 1) for correlations of *ρ* = 0.9, 0.7, and 0.6 are 14 (1 step), 33 (2 steps), and 70 (3 steps), respectively.

Fig 5 also illustrates the large effect of correlations between serial decision variables (Fig 1B). When the correlation between serial decision and reference variables is high, attrition rates at steps later in the process tend to be low, because a candidate that passes through one decision step is likely to pass the next. In Fig 5, stringency, the proportion of the starting candidates that exceed each decision threshold and the reference threshold, is constant across the different conditions. However, there are large differences in overall attrition rates, expressed as the number of candidates screened per *TP* in Fig 5B. When the correlation coefficients are 0.9, a four step process would screen 22 candidates per *TP*, and the *FDR* would be a mere 9% (*PPV* = 91%). When the correlation coefficients are 0.3, a four step process would screen ~739 candidates per *TP*, and the *FDR* would be 52% (*PPV* = 48%).

**Fig 6:**
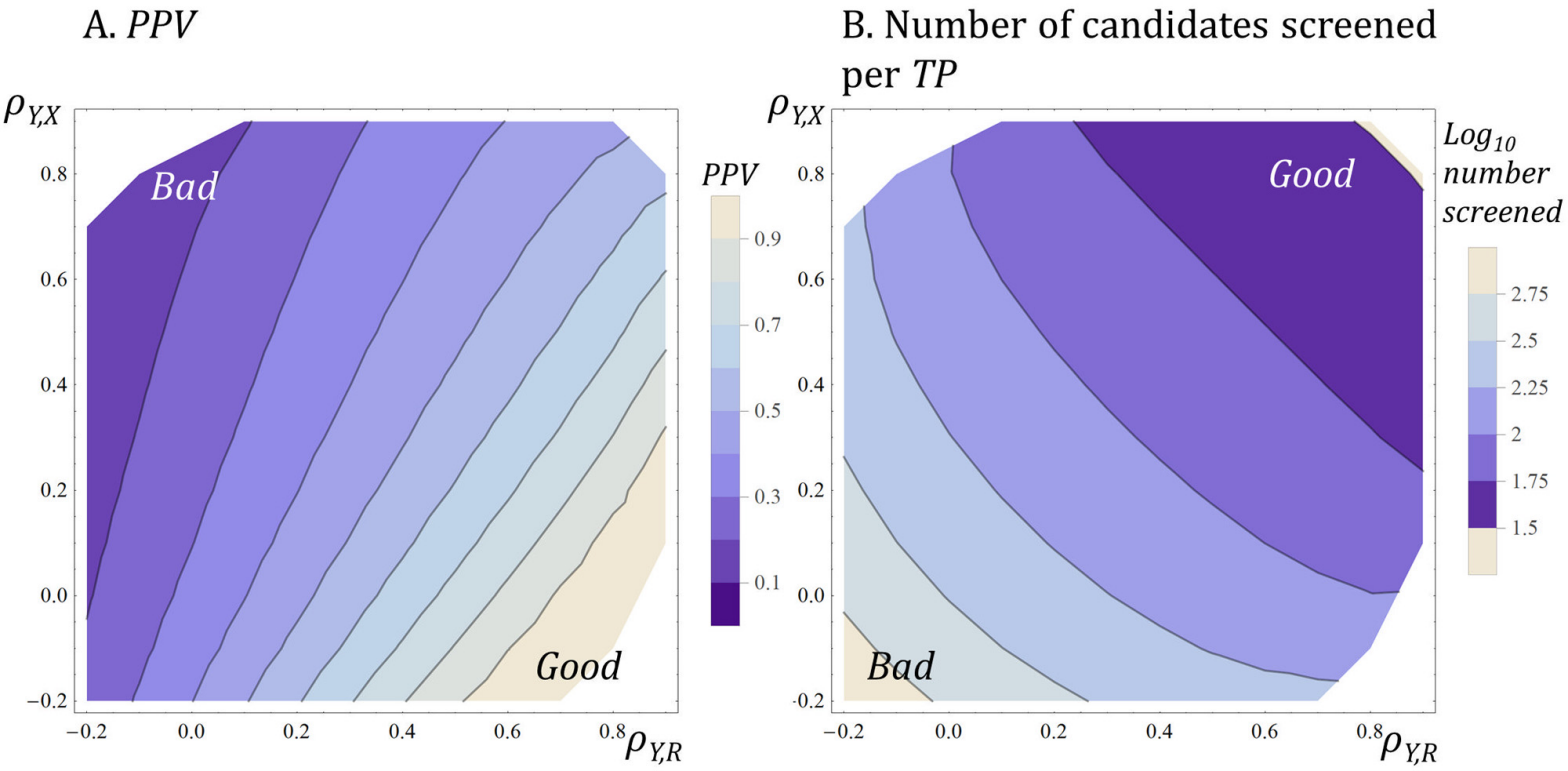
Decision performance as correlations between decision variables change. The first decision variable was *X*, and the correlation coefficient between *X* and *R*, *ρ*_*X*,*R*_, was held constant at 0.5. The second decision variable was *Y* which varied in terms of its correlation with *X* (*ρ*_*Y*,*X*_, vertical axes) and with reference variable *R* (*ρ*_*Y*,*R*_, horizontal axes). Some regions of the graphs are empty because certain combinations of correlation coefficients cannot coexist. The top decile of candidates in the starting set exceed each decision threshold and the reference threshold (i.e., P(*X* ≥ *x*_*t*_) = P(*Y* ≥ *y*_*t*_) = P(*R* ≥ *r*_*t*_) = 0.1). (A) shows *PPV*. Lighter shades indicate higher *PPV*. *PPV* increases as *ρ*_*Y*,*R*_ increases and as *ρ*_*Y*,*X*_ declines. The use of *Y* may depress *PPV* if *Y* is highly correlated with *X* while having a low correlation with *R*. (B) shows the number of candidates screened per *TP*. Darker shades indicate fewer candidates per *TP*. Note the log_10_ colour scale. The number increases as *ρ*_*Y*,*R*_ declines and as *ρ*_*Y*,*X*_ declines.

Fig 6 illustrates some of the effects on decision performance of varying the correlation, *ρ*_*Y*,*X*_, between two decision variables, *X* and *Y*, and varying the correlation, *ρ*_*Y*,*R*_, between decision variable *Y* and the reference variable *R*.

Fig 6 shows, first, that *PPV* increases and the number of candidates screened per *TP* decreases with an increase in *ρ*_*Y*,*R*_. Things are better if the second decision variable is highly correlated with *R*. This is no great surprise.

Second, and less intuitively obvious perhaps, is the fact that *PPV* increases but the screening effort also increases as the correlation between the two decision variables, *ρ*_*Y*,*X*_, decreases. The effect of changes in *ρ*_*Y*,*X*_, independent of the degree to which either measure correlates with *R*, can be powerful (vertical axes in Fig 6). This is why counter-screening works [75] [76] and why absorption, distribution, metabolism, and excretion (ADME), toxicology, and efficacy measures, are much more informative when combined. However, the cost of combining variables that are uncorrelated with each other can be a large increase in the number of candidates screened per *TP*, because few candidates will score well on several independent measures.

It may also surprise some that the addition of a second decision variable and classifier can **depress** *PPV*. This occurs if the second decision variable is highly correlated with the first, but has a low correlation with the reference variable, *R*. In practical terms, this shows that PMs cannot be regarded as ‘valid’ simply because their output correlates with the output of other PMs. It may often make sense to seek out and add PMs that have face validity versus *R* but which yield decision variables that have a low correlation with other decision variables.

## Discussion

### 1. The Exhaustion and Abandonment of High PV Models

This paper was motivated by a desire to explain “*Eroom’s Law*” [1]: The approximate halving every 9 years between 1950 and 2010 in the number of new drug molecules approved by the FDA per billion dollars of inflation-adjusted R&D investment by the drug industry, in the face of huge gains in knowledge and in brute-force efficiency.

One standard explanation for Eroom’s Law is that the “low hanging fruit” have been picked. We and others have been critical of such explanations [77] [1]. First, they generally leave the nature of the fruit undefined (but there are exceptions [78]). Second, such explanations may underestimate the difficulty of historical discoveries [77] [24] [1]. Third, drugs that come to market reduce the incremental economic and therapeutic value of undiscovered or unexploited therapeutic candidates without making such candidates harder to discover *per se*. This is the so-called “*better than the Beatles problem*” [1]. Fourth, low hanging fruit explanations risk tautology, because they use the efficiency of R&D as the measure of the height at which as-yet-unpicked fruits are hanging [1].

However, the analyses in this paper suggest what may be an important kind of fruit. Changes in the PV of decision variables that many people working in drug discovery would regard as small and/or unknowable (i.e., a 0.1 absolute change in correlation coefficient versus clinical outcome) can offset large (e.g., 10 fold or greater) changes in brute-force efficiency. Furthermore, the benefits brute-force efficiency decline as the PV of decision variables declines (left hand side of both panels in Fig 4). It is our hypothesis, therefore, that much of the decline in R&D efficiency has been caused by the progressive exhaustion of PMs that are highly predictive of clinical utility in man. These models are abandoned because they yield successful treatments. Research shifts to diseases for which there are poor PMs with low PV [78]. Since these diseases remain uncured, people continue to use bad models for want of anything better. A decline in the average PV of the stock of unexploited screening and disease models (PMs) can offset huge gains in their brute-force power (Fig 4).

We also suspect that there has been too much enthusiasm for highly reductionist PMs with low PV [26] [79] [25] [80] [81] [74] [82]. The first wave of industrialized target-based drug discovery has been, in many respects, the embodiment of such reductionism [1] [83] [84] [74]. The problem is not necessarily reductionism itself. Rather, it may be that good reductionist models have been difficult to produce, identify, and implement [85] [82], so there has been a tendency to use bad ones instead; particularly for common diseases, which tend to have weak and/or complex genetic risk factors [86] [83] [87]. After all, brute-force efficiency metrics are relatively easy to generate, to report up the chain of command, and to manage. The PV of a new screening technology or animal PM, on the other hand, is an educated guess at best. In the practical management of large organisations, what is measureable and concrete can often trump that which is opaque and qualitative [65], even if that which is opaque and qualitative is much more important in quantitative terms.

We note here what appears to be a real uptick in drug approvals from ~2012. We think this reflects the ability of modern methods to increase the PV of models for specific cancer subtypes and other rare diseases with strong and simple genetic risk factors [83]. Molecular diagnostics, for example, make it easier to match reductionist PMs’ “domains of validity” with human pathology in these rare diseases.

The history of drug discovery also points to the importance of PV over throughput. During the Golden Age of therapeutic innovation [24], some drug R&D resembled phenotypic screening in man. Throughput was low, mechanistic understanding was limited, experimental design and conduct (e.g., randomisation, blinding, etc.) often left much to be desired when compared with modern standards, but the decision variables (i.e., observations of clinical responses in humans) had high PV for the reference variable (i.e., clinical responses in humans) [24] [88] [89] [69]. Even in modern times, “field discovery” by practicing physicians appears to be a major, if under-appreciated, source of pharmacological innovation [90] that occurs in the face of remarkably low drug throughput. There are, after all, only in the order of 1,000 approved drug molecules whose effects in man can be observed by physicians [91].

We hypothesize that the rate of creation of valid and reliable PMs may be the major constraint on industrial R&D efficiency today [16] [92]. If this hypothesis is even partly true, it points to a mismatch between those areas where valuable intellectual property is relatively easy to secure (e.g., novel chemical structures) and those areas where incremental investment would be most useful for the wider good (i.e., good PMs for poorly treated conditions).

### 2. The Reproducibility Crisis and Predictive Validity

It is common to think of validity and reproducibility or reliability as different things (Table 1). After all, the existence of reference tests against which the output of a model may or may not correlate is irrelevant for whether or not the results of that model are consistent when it is repeatedly applied. However, as with Eroom’s Law [1] (above), we hypothesize that the academic reproducibility crisis [13] [92] [93] [94] could reflect the abandonment of models with high PV, for reasons of exhaustion and/or scientific fashion.

**Fig 7:**
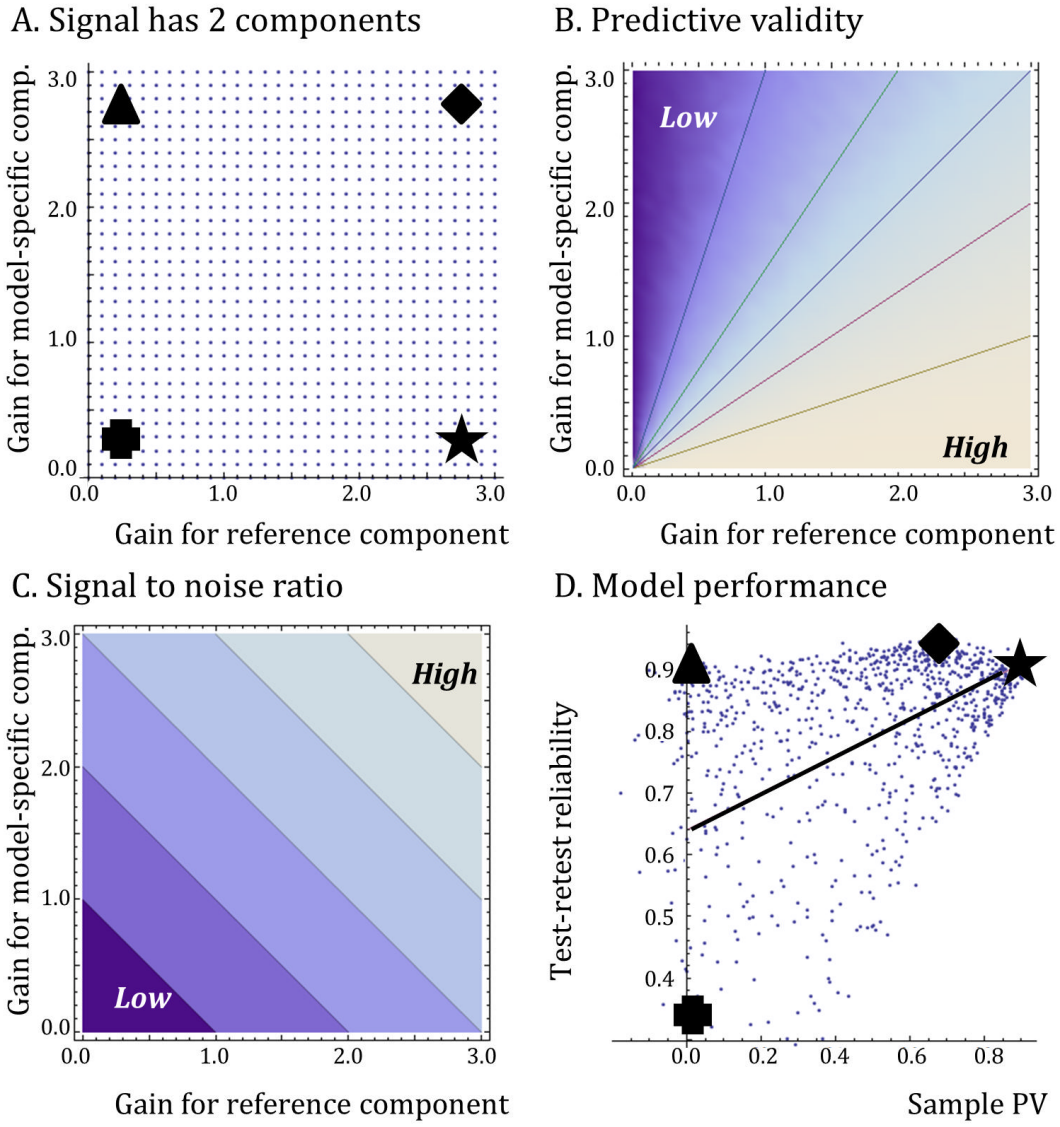
Link between validity and reproducibility across a set of screening and disease models. The figure shows the results of a Monte Carlo simulation (see S1 File for code). (A) Each small point represents one simulated screening or disease model (PM). When testing therapeutic candidates, each PM yields an expected signal which is the sum of two components. The first component is the signal from the reference test multiplied by a gain parameter (horizontal axis). The second component is a model-specific signal, whose gain is shown on the vertical axis. This component can also be thought of as systematic model-specific bias. It is real, but it tells us nothing about the reference test. (B) Each model’s PV is determined by the relative strength of the reference component versus the model-specific component of the signal. PV is high when the reference component is much larger than the model-specific component of the signal. This is because the output of the PM will correlate with the reference test when its signal is dominated by the reference signal. (C) Each PM’s signal to noise ratio increases with the sum of the reference component and the model-specific component. (D) Each point represents the performance of one of the models in Panel A., in two simulated experiments that include sampling and measurement noise. The horizontal axis shows the results of the first experiment. It is sample predictive validity (the correlation coefficient between the output of the model and the output of the reference test for a sample of therapeutic candidates). The vertical axis is the second experiment. It is test-retest reliability using the same sample of therapeutic candidates (calculated as the correlation coefficient between the results of the test and retest). The symbols (star, diamond, triangle, and cross) show how the space in (A) maps onto the space in (D). The line in (D) shows the best fit for the linear regression between sample PV and test-retest reliability. For the simulation shown, we sampled 400 therapeutic candidates for each PM. Both the reference and model-specific components of PM’s signal were drawn from a normally distributed random variable, whose mean was zero and whose standard deviations were equal to the respective gains on the horizontal and vertical axes of (A) to (C).

Our argument is illustrated in Fig 7. Imagine retiring the models with high PV, which are those at the right hand end of the horizontal axis in Fig 7D. These are the models most likely to give answers that are obvious and useful, thus rendering themselves redundant. As the high PV models are progressively retired, the average signal to noise ratio and the average test-retest reliability of the remaining stock of models falls (regression line and vertical axis, Fig 7D). With a lower signal to noise ratio in the remaining stock, the play of chance [13] [29] [72] and professional biases [95] [96] [94] can start to exert more visible effects on the quality of the scientific literature.

### 3. Improving Predictive Validity

If one accepts the main conclusion we draw from this paper, that PV has a very powerful effect on R&D decision quality and productivity, one is left–as our reviewers pointed out–with a difficult but important question: *“Can you estimate PV prospectively*, *or improve the PV of models in as-yet uncharted therapy areas*?*”*

Measuring and managing PV is difficult for several reasons. It is impossible to test a large number of candidates across multiple PMs and then in man. It is impossible, therefore, to measure PV with high precision, even in mature therapy areas. Furthermore, by the time a therapy area is mature, there is less reason to invest in calibrating PMs. This means PV estimates will be, at best, educated guesses. None the less, we do have some suggestions.

First, we suspect that experienced scientists often have an intuition about the PV of the models at their disposal, but today make the wrong trade-off between PV and unit cost, throughput, convenience, or scientific fashion. They should give more weight to their own expert judgement of PV, even if this means screening an order of magnitude fewer therapeutic candidates or writing far fewer papers. Funding decisions must support this behaviour by prioritising the quality of argumentation around PM choice and PV.

Second, we suspect that much useful information on PV is neither captured, nor systematized, nor communicated to those making R&D decisions. Between [97], and even within [71] [98], biomedical disciplines, validity-related and reliability-related terminology and concepts are inconsistently applied. This means that groups of people who work together (e.g., when reviewing grant applications or project proposals) should discuss and agree a *lingua franca* for validity and reliability-related concepts. Here we have been struck by work on data pedigree [99] in the field of environmental risk assessment. Environmental policy decisions are sometimes science-based but often politically contentious. Therefore, it is important to communicate the pedigree of models along with the results that they yield. Pedigree would consider factors such as the extent to which the model is based on well-established theoretical frameworks (similar to the concept of “construct validity” [100]); etc. We recommend work to develop and apply concepts of data pedigree to the results derived from screening and disease models.

There is a wonderful term, “domains of validity” that is widely used in physics but which used little, if at all, in biomedical research. It refers to the parameter space within which a model is valid. For example, classical mechanics has a large and clear domain of PV, which includes the trajectory of a jumping flea, the orbits of the moons of Neptune, but not way stars’ gravity “bends” light, nor the way electrons move around atoms. People know this and apply classical mechanics accordingly. Efficient drug R&D requires domains of PV at each step that extend to clinical utility in man (Fig 1B). PMs that may be competently reported and reproducible in a narrow technical sense can fail because their domains of validity are too narrow [101]; they “work”, but are not usefully generalizable. Biomedical journals should therefore require that authors sketch out and justify the domains of validity of the PMs they use.

Third, and finally, we recommend investment in empirical studies of the PV of screening and disease models across a diverse set of diseases for which we have at least some approved drugs. This should include analysis of the correlations between of the outputs of different, preferably sequential, PMs, and qualitative analyses of the PMs themselves, and of how they are used to make R&D decisions. There is already work in this general area (e.g., references [80] [102] [56] [15] [14] [16] [32] [101] [103] [104] [62] [105] [106] [107]), but there is not enough. We also suggest the production of standard collections of drugs and chemical probes that can be used, therapy area by therapy area, to cross-calibrate PMs [101]. The long-run aim should be to derive and back-test “meta-models”—qualitative or narrative in the first instance (e.g., references [80] [102])—that are themselves predictive of screening and disease models’ predictive validity.

## Supporting Information

S1 File Mathematica 9.0 code to reproduce analyses in Figs 2–7. (ZIP)

S2 File Analysis of alternative probability density functions. (PDF)
