# Supplementary material for: When Quality Beats Quantity: Decision Theory, Drug Discovery, and the Reproducibility Crisis
Source: PLoS One. 2016 Feb 10;11(2):e0147215. doi: 10.1371/journal.pone.0147215 (PMC4749240; doi:10.1371/journal.pone.0147215)

S2 File. Scannell & Bosley. Alternative Probability Density Functions

For the results presented in the main paper, we assumed that the probability density of candidate molecules within measurement space is a multivariate Normal distribution determined by the correlation matrix between the decision variable Y and the reference variable R. We believe the results are generalizable to many, but not all, alternative distributions. We would expect to find similar results under the following conditions: First, when $P(R \geq r_t) \leq 0.1$ and $P(Y \geq y_t) \leq 0.1$; second, when the relationship between Y and R is unimodal (at least in the tails of distribution where the search for yeses and positives is taking place); and third, in distributions where being at an extreme value on y is not strongly associated with being at an extreme value on r "quasi-independent" of the correlation coefficient. In such distributions, the correlation coefficient, which is influenced by the bulk of the probability mass as well as the tails of the distribution, can be a poor guide to strong associations that exist at extreme values of y and r (i.e., the regions that we are searching for *yeses* and for *positives*).

Some results derived using alternative probability density functions are shown below. The figures below should be compared with Fig. 2 and Fig. 4 in the main paper. Figures A2 and A3, derived from bivariate Normal PDFs, are identical to Figure 4A and 4B in the main paper. Figures B2 and B3, derived from bivariate Lognormal PDFs, are identical to A2 and A3 (barring some of the practicalities of numerical integration). In Figures C2 and C3, which are derived from a bivariate Student's t-distribution, with the degrees of freedom parameter set at $\nu = 5$, the correlation ρ (representing the predictive validity of the decision variable) becomes much less important, and throughput becomes much more important, than in the analyses derived from the Normal and Lognormal PDFs. In figures D2 and D3, based on a uniform PDF, the converse is true. PPV is much more sensitive to ρ (predictive validity) relative to y_t (throughput) than is the case in either the Normal or Lognormal PDFs. In panel D3, for example, each line represents a different level of ρ. The graph shows that PPV is relatively insensitive to y_t across several orders of magnitude.

We suggest that the parameter ρ in the case of the heavy-tailed Student's t-distribution is sensitive to relatively small quantities of probability mass with extreme scores on both y and r. The opposite occurs in the uniform distribution. Here the correlation is mainly driven by points that lie outside of the extremes of the distribution, so the set of items from the sample that exceeds y_t contains relatively few items that exceed r_t.

On a more practical note, these observations suggest that the efficiency of screening strategies will be sensitive to the form of the distribution of molecules in measurement space, as well as to

the correlations that exist between the decision variables and between the decisions variables and the reference variable.

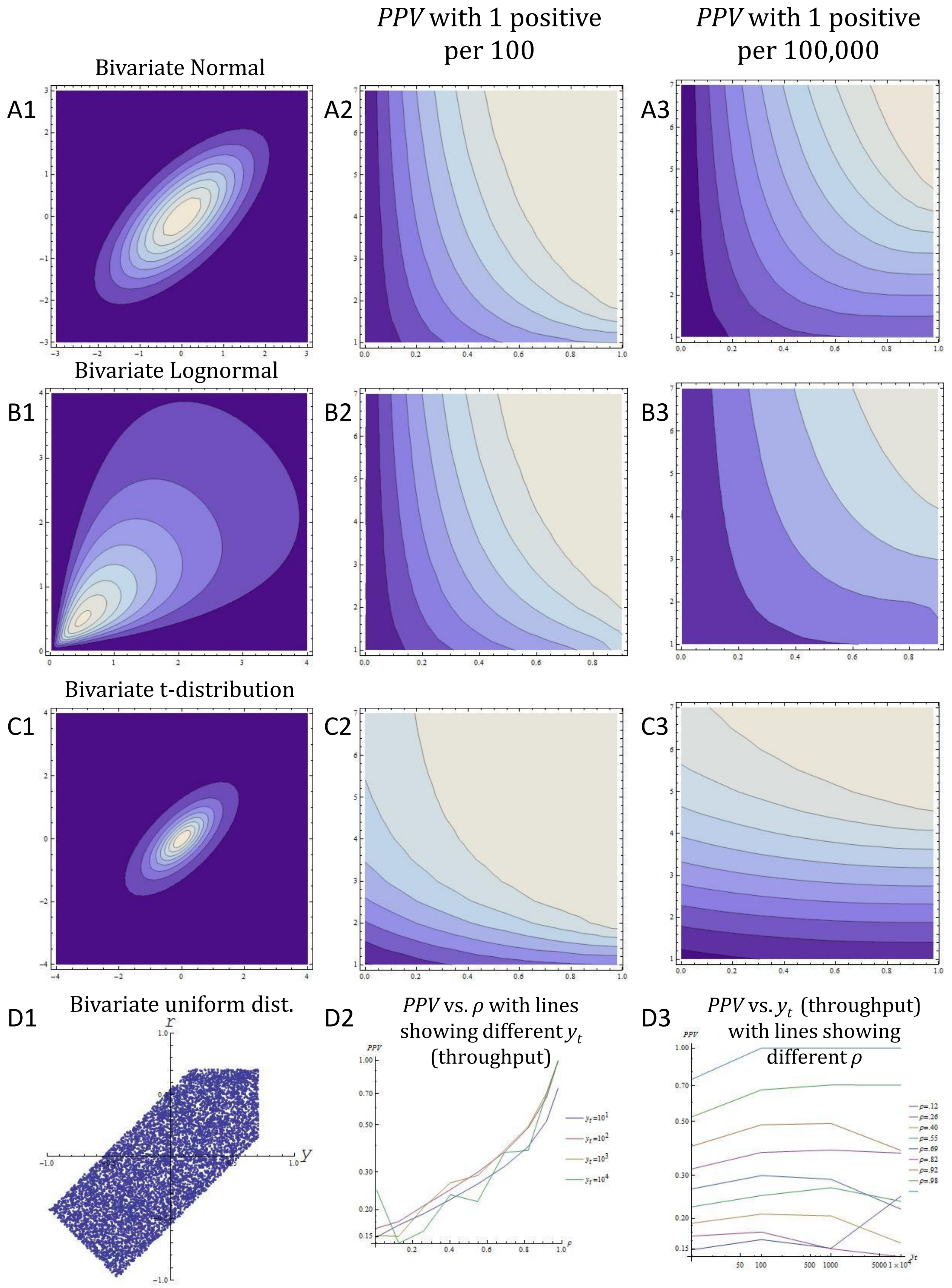

Supplement: S2 File — (PDF) [file pone.0147215.s002.pdf]
